# International Legal Approaches to Neurosurgery for Psychiatric Disorders

**DOI:** 10.3389/fnhum.2020.588458

**Published:** 2021-01-13

**Authors:** Jennifer A. Chandler, Laura Y. Cabrera, Paresh Doshi, Shirley Fecteau, Joseph J. Fins, Salvador Guinjoan, Clement Hamani, Karen Herrera-Ferrá, C. Michael Honey, Judy Illes, Brian H. Kopell, Nir Lipsman, Patrick J. McDonald, Helen S. Mayberg, Roland Nadler, Bart Nuttin, Albino J. Oliveira-Maia, Cristian Rangel, Raphael Ribeiro, Arleen Salles, Hemmings Wu

**Affiliations:** ^1^Faculty of Law, University of Ottawa, Ottawa, ON, Canada; ^2^Center for Ethics & Humanities in the Life Sciences and Dept. Translational Neuroscience, Michigan State University, East Lansing, MI, United States; ^3^Department of Neurosurgery, Jaslok Hospital and Research Center, Mumbai, India; ^4^Department of Psychiatry and Neurosciences, Faculty of Medicine, Université Laval, Quebec City, QC, Canada; ^5^CERVO Brain Research Center, Center Intégré Universitaire en Santé et Services Sociaux de la Capitale-Nationale, Quebec City, QC, Canada; ^6^Weill Cornell Medical College, Consortium for the Advanced Study of Brain Injury, Weill Cornell and the Rockefeller University, New York, NY, United States; ^7^Solomon Center for Health Law & Policy, Yale Law School, New Haven, CT, United States; ^8^Laureate Institute for Brain Research, Tulsa, OK, United States; ^9^Harquail Center for Neuromodulation, Sunnybrook Research Institute, Division of Neurosurgery, Sunnybrook Health Sciences Center, University of Toronto, Toronto, ON, Canada; ^10^Asociación Mexicana de Neuroética, Mexico City, Mexico; ^11^Section of Neurosurgery, Max Rady College of Medicine, University of Manitoba, Winnipeg, MB, Canada; ^12^Neuroethics Canada, Division of Neurology, Department of Medicine, University of British Columbia, Vancouver, BC, Canada; ^13^Departments of Neurosurgery, Neurology, Psychiatry and Neuroscience, Icahn School of Medicine at Mount Sinai, New York, NY, United States; ^14^Division of Neurosurgery, Harquail Center for Neuromodulation, Sunnybrook Health Sciences Center, University of Toronto, Toronto, ON, Canada; ^15^Division of Neurosurgery, Faculty of Medicine, BC Children's Hospital, University of British Columbia, Head, Vancouver, BC, Canada; ^16^Departments of Neurology, Neurosurgery, Psychiatry and Neuroscience, Icahn School of Medicine at Mount Sinai, New York, NY, United States; ^17^Peter A. Allard School of Law, University of British Columbia, Vancouver, BC, Canada; ^18^Neurosurgeon, Katholieke Universiteit (KU) Leuven, Universitair Ziekenhuis (UZ) Leuven, Leuven, Belgium; ^19^Champalimaud Research and Clinical Center, Champalimaud Center for the Unknown, Lisbon, Portugal; ^20^NOVA Medical School, NMS, Universidade Nova De Lisboa, Lisbon, Portugal; ^21^Department of Innovation in Medical Education, Faculty of Medicine, University of Ottawa, Ottawa, ON, Canada; ^22^Faculty of Law, University of Ottawa, Ottawa, ON, Canada; ^23^Center for Research Ethics and Bioethics, Uppsala University, Uppsala, Sweden; ^24^Department of Neurosurgery, Second Affiliated Hospital, Zhejiang University School of Medicine, Hangzhou, China

**Keywords:** neuroethics, regulation, law, deep brain stimulation, psychosurgery, neurosurgery for psychiatric disorders

## Abstract

Neurosurgery for psychiatric disorders (NPD), also sometimes referred to as psychosurgery, is rapidly evolving, with new techniques and indications being investigated actively. Many within the field have suggested that some form of guidelines or regulations are needed to help ensure that a promising field develops safely. Multiple countries have enacted specific laws regulating NPD. This article reviews NPD-specific laws drawn from North and South America, Asia and Europe, in order to identify the typical form and contents of these laws and to set the groundwork for the design of an optimal regulation for the field. Key challenges for this design that are revealed by the review are how to define the scope of the law (what should be regulated), what types of regulations are required (eligibility criteria, approval procedures, data collection, and oversight mechanisms), and how to approach international harmonization given the potential migration of researchers and patients.

## Introduction

The history of neurosurgery for psychiatric disorders (NPD) is one of extremes running from celebration and expanded use to backlash and public condemnation. António Egas Moniz was awarded the 1949 Nobel Prize for the prefrontal leucotomy, and a form of this, known more often as the lobotomy, was pursued particularly but not exclusively in the US until the late 1960s. At this point, the availability of effective antipsychotic drugs, along with changes in social attitudes to psychiatry, the rise of the civil rights movement, and concerns about the application and side effects of the lobotomy all contributed to a strong shift away from what was then usually called psychosurgery (Pressman, 1998; Robison et al., 2012; Caruso and Sheehan, 2017). Cultural products such as Suddenly Last Summer (Williams, 1958; Spiegel and Mankiewicz, 1959), One Flew over the Cuckoo's Nest (Kesey, 1962; Douglas et al., 1975), and Hombre Mirando al Sudeste (Pflaum et al., 1986) reflect different societies' concerns in the 1960s to 1980s with the role of psychiatry, physical treatments involving the brain, and state control of behavior. An unusual law reflecting this kind of concern is the Utah Code provision stating that it is a criminal offense to give psychiatric treatment, including “lobotomy or surgery” to any person “for the purpose of changing his concept of, belief about, or faith in God”^1^.

During this period of controversy, the US National Commission for the Protection of Human Subjects of Biomedical and Behavioral Research conducted an evaluation of psychosurgery (1977). The National Commission, also known for the (United States National Commission for the Protection of Human Subjects of Biomedical Behavioral Research, 1979), issued a generally favorable report and provided guidelines for the ethical use and regulation of psychosurgery (Fins, 2003). Also in this period, multiple legal jurisdictions enacted their own laws that specifically regulate NPD. These laws often used the term psychosurgery, and some defined the term to include non-ablative interventions such as deep brain stimulation (e.g., Ontario, Canada). In some jurisdictions, NPD-specific laws have been recently amended, demonstrating continued regulatory interest in the topic. However, many locations do not have NPD-specific laws, leaving the field of practice to be regulated under general laws pertaining to medicine [e.g., Mexico, Argentina, Manitoba (Canada)].

Today, brain interventions intended to restore functions that are disrupted in psychiatric conditions are rapidly evolving. The technologies available, and the scope of targeted medical conditions potentially suitable for these interventions, are expanding swiftly. Rapid technological evolution presents a major challenge for legal systems. Poorly designed laws may impede or distort that evolution, and may also fail to achieve their key objectives of, for example, protecting the interests of vulnerable patients, promoting the public interest, and encouraging beneficial innovation. Another challenge for democratic legal systems is how to ensure that the perspectives and experiences of a broad range of stakeholders are reflected in the compromises that are struck in the eventual laws (statutes, regulations, and common law). The interests of patients, caregivers, researchers, medical practitioners, device manufacturers, and the public may diverge, and the laws adopted must appropriately balance these interests, although structural and political factors mean that certain interests may dominate.

The existing legal frameworks are patchy and have failed to keep up with scientific, technological and social change. The scope of applicability of the existing NPD-specific laws is often unclear and many are partly obsolete, reflecting old science, methods of intervention and social currents of decades past. The laws also reflect mind-brain dualism, with many restricted to interventions to treat mental illnesses, while excluding similar interventions intended to treat what are categorized instead as neurological or brain illnesses. The international discussion amongst clinical experts recognizes a need for some form of guidelines for the field (Wu et al., 2012; Nuttin et al., 2014; Bari et al., 2018; Doshi et al., 2019). Occasional calls for mandatory forms of regulation have been made by members of the relevant clinical community (Wu et al., 2012; Visser-Vandewalle, 2014).

Rules come in many forms, both legal and non-legal. Consensus guidelines produced by authoritative professional bodies, policies adopted by hospitals or medical regulators, and self-regulatory procedures created by groups of medical practitioners are examples of rules that may guide practice even though they do not constitute formal legal rules and principles developed by judges or enacted by government bodies. Each of these forms has pros and cons in terms of the ease with which they may be prepared and amended over time, their enforceability, their geographical applicability, and other features. It is not immediately clear which form would be best suited to regulating NPD, and different approaches may be preferable depending upon the type of intervention and the particular society and legal culture. In some jurisdictions clinicians have created forms of self-regulation. For example, psychiatrists and neurosurgeons in the Netherlands formed a review board in the 1970s that went on to review cases from Belgium and the Netherlands. It was endorsed by the Dutch Health Council as a “good example of self-regulation” (Cosyns et al., 1994). This body continues to operate (Gabriëls et al., 2008). Cultures and jurisdictions may also differ in their views of what those regulations should say–reflecting variation in underlying conceptual and ethical views as well as the social and economic realities that shape regional reactions to NPD.

Against this backdrop, six key questions are raised:

1. Is there a need for specific rules addressing NPD, or can this be left to more general rules applicable to medical practice and research, and/or to mental health legislation?

If specific rules addressing NPD are needed, then:

(2) Which forms of current and anticipated intervention require specific rules (i.e., is “NPD” as a category for regulation too broad or narrow a target)?
(3) Should the rules be the same for all forms of NPD, or is a different approach appropriate for particular interventional techniques (e.g., ablative or not, requiring surgical incision or not, investigational or established), indications, patient populations (e.g., children, incapable adults, or other vulnerable populations such as institutionalized people)?
(4) What form should the rules take (e.g., legal statutes, professional guidelines) and what form of oversight and enforcement is best (e.g., oversight committees, licensing bodies or tribunals; prior authorization of procedures or ex post reporting; advisory or mandatory decisions)?
(5) What issues should the rules address (e.g., eligibility of patients, consent procedures, reporting requirements, training and suitability of medical personnel or centers)?
(6) Should these rules be harmonized across different legal jurisdictions? To what extent is this possible, and what is the optimal process to achieve this?

We do not attempt to answer all of these questions in this article. Instead, we set the groundwork for answering them by offering a structured overview and assessment of existing legislation that specifically addresses NPD from a range of international jurisdictions. The overview illustrates the kinds of rules, procedures and enforcement mechanisms that have been enacted, and offers commentary on their current adequacy.

## Method and Limitations

### Objective

The objective of this work is to examine a selection of NPD-specific legislation enacted around the world, and to consider the strengths and weaknesses of the approaches taken in those laws.

### Data Collection

Legal systems vary greatly around the world, encompassing the civilian (i.e., Roman) tradition, common law tradition, customary and religious law, and mixed systems. We focus on primary legislative texts (e.g., statutes, codes, and decrees) enacted by government bodies as opposed to judicial decisions or doctrine. We took a three-tiered approach to selecting legislation for consideration in this study. First, drawing on the legal expertise within the authorship group, we conducted a comprehensive search for NPD-specific legislation in Canada (13 provinces and territories) and the United States (50 states). In a second step, we broadened this inquiry by searching the laws of certain Western Commonwealth countries (Australia, New Zealand, Ireland, Scotland, and England). We selected these because these countries reflect a common law tradition consistent with the legal expertise of the primary author. Finally, we drew on the full group of co-authors who collectively represent eight countries in South Asia, East Asia, South America, North America and Europe to help us to find legislation from their jurisdictions. This three-stage effort at data collection furnished a robust cross-section of statutes from which to comment on some of the ways that NPD legislation has been approached, but it does not necessarily represent all NPD-specific legislation that may exist around the world.

### Analysis

We employed several analytical methods in this work. First, the rules of statutory interpretation includes principles according to which the meaning of the written text and its application in concrete scenarios is determined (Solan, 2010). In interpreting the statutes, we relied primarily on the “plain meaning” of the words used, as well as the inclusion of definitional or interpretive aids within the statutes themselves, and the placement of the provisions within the broader structure of the statute. Second, we conducted a thematic qualitative legal analysis, organizing the contents of the statutes into themes that we found to be repeated across multiple statutory examples. We took note of unusual approaches as well, given our interest in capturing a range of potential approaches to regulating NPD. Our author group included international and interdisciplinary representation from clinical neuroscience, psychiatry, neurosurgery, neurology and neuroethics, and we evaluated the statutory language in light of this medical and scientific expertise to determine the suitability of the statutory language to the field and its potential future evolution.

### Results and Presentation

Our analysis revealed several cross-cutting themes raised by most or all of the examples of NPD-specific statutes considered here. We organized these as follows: (1) definition of the field and scope of the law (section Legal Definitions of NPD), (2) specific rules prescribed for the field (sections Regulations–Prohibitions Applying to Certain Procedures or Populations, Regulations–Independent Approval Procedures, and Regulations–Data Collection Requirements). The international and interdisciplinary authorship group also identified certain matters that were not addressed in the statutes we considered; we decided to include discussion of those following the summary of the statutes we examined (sections Experimental Forms of NPD and Regulatory Variation and the International Movement of Patients).

### Limitations

A limitation of this work is that we cannot provide a comprehensive picture of all of the law related to NPD around the world or within any one jurisdiction, each of which has its own legal structure and broader body of laws that may apply concurrently with any NPD-specific laws. In addition, each jurisdiction has its own principles of legal interpretation, and judicial decisions interpreting the legislation, which we do not examine here. Therefore, our review should not be relied upon as an authoritative statement of the relevant law, which should be sought from locally licensed lawyers if needed. Instead we have collected a broad sample of NPD-specific laws around the world in order to identify patterns in these laws.

As we focus in detail on NPD-specific laws, we have not included laws regulating electroconvulsive therapy (ECT), although these also exist in many jurisdictions. Also we have focused solely on formal laws, and have not included consensus guidelines or any other forms of self-regulation.

Finally, many NPD procedures remain investigational, and so the rules applicable to medical research in human subjects would apply along with any NPD-specific laws. We do not review those rules here, and it is important to note that the NPD-specific laws usually ignore the distinction between interventions that constitute established therapies and those that remain experimental.

## Legal Challenges Posed by Evolution in Psychiatric Neurosurgery

The existing and potential future range of technologies available for neurosurgery and neuromodulation for psychiatric disorders is varied and rapidly evolving. This complicates an effort to determine when regulation is needed, what form of regulation is advisable, and how to define the scope of the laws. As will be discussed later, the laws that address specific psychiatric brain interventions focus primarily on one or more of the following: ablative neurosurgery, non-ablative surgical interventions such as deep brain stimulation (DBS), and electroconvulsive therapy (ECT). An important question is whether this focus is appropriate given the evolution in techniques of functional brain intervention. Here, we briefly survey the evolving range of techniques of psychiatric neurosurgery. Although not all of these would fit within the current laws governing NPD, it is important to consider the broader potential range of interventions in deciding on what the optimal law going forward would be.

Ablative neurosurgery can be performed using incisional and incision-less methods of accessing and lesioning targeted brain tissue [e.g., stereotactic radiosurgery, magnetic resonance image-guided laser interstitial thermal therapy (LITT) and magnetic resonance image-guided focused ultrasound thermal ablation (MRgFUS)] (Franzini et al., 2019). Ablative procedures continue to be provided on a small scale for serious treatment-resistant psychiatric disorders such as major depressive disorder (MDD) and obsessive compulsive disorder (OCD) (e.g., anterior cingulotomy, capsulotomy) (Nuttin et al., 2014). In most developed countries, NPD has shifted from ablative procedures to the neuromodulatory approach of DBS (Hariz and Hariz, 2013), although this is not the case for resource-poor contexts where access to medication, psychotherapy, and more expensive DBS is limited (Nuttin et al., 2014). DBS is being investigated for a broad and increasing range of neurological and psychiatric problems including MDD, OCD, addiction, Tourette's syndrome, eating disorders, pain, disorders of consciousness, aggression, post traumatic stress disorder (PTSD), and dementia (Nuttin et al., 1999; Mayberg et al., 2005; Schiff et al., 2007; Lee et al., 2019). Other forms of psychiatric neuromodulation requiring surgical access include epidural and subdural stimulation (Tronnier and Rasche, 2013, p. 343). Surgical neuromodulation for psychiatric conditions is not always applied directly to the brain but can also be delivered via the peripheral nervous system, as with vagus nerve stimulation. Multiple techniques for externally applied neuromodulation such as transcranial electric stimulation (tES) and transcranial magnetic stimulation (TMS) are now being explored. Some of these are in use clinically to treat certain psychiatric disorders and they are being actively explored for others (Cristancho et al., 2013; George et al., 2013; Lefaucheur et al., 2017, 2020). In the future, externally applied techniques may allow for the modulation of more precisely delimited structures deeper within the brain through electrical field interference (Grossman et al., 2017) or low-intensity focused ultrasound (Di Biase et al., 2019).

Another substantial technological change in neuromodulation is the move toward responsive or adaptive methods that monitor brain activity and deliver stimulation only when needed rather than continuously. This technique, known as responsive neurostimulation (RNS) is approved for adult epilepsy (FDA, 2020), and is being explored for broader use. The integration of machine learning to optimize modulation algorithms, as well as the incorporation into the algorithms of a broad range of markers to identify the need for neuromodulation (Hell et al., 2019) could produce more powerful, flexible, and complex neuroprostheses for the management of dysfunctional brain states.

Technological evolution may also cause some forms of treatment like psychotropic drug therapy that have not fallen within NPD laws in the past to fall within the scope of the laws. Methods to deliver drugs directly to the brain via a surgical approach (in a manner analogous to existing forms of intrathecal drug delivery) are possible in future (Fowler et al., 2020), and when used to address psychiatric disorders would constitute a form of neurosurgery that would fall outside some existing legal definitions of NPD but within others.

All of this means that laws may become ambiguous, obsolete, overly broad or under-inclusive over time as methods of intervention change. This poses a significant challenge to designing appropriate and useful regulation.

Multiple factors are relevant to the question whether some form of regulation is required and if so what it should say. Factors relevant to the method of intervention include safety, reversibility, effectiveness, side effects, and practicality. While all ablative techniques are intended to produce irreversible brain lesions, non-incisional ablative techniques such as focused ultrasound or radiosurgery may avoid risks like infection that are associated with incisional techniques. Neuromodulation techniques differ in whether they (a) are general, diffuse or precisely targeted in their stimulatory effects, (b) are able to affect superficial brain tissue or deeper structures, (c) require surgical incisional access or are applied externally, (d) are applied directly to the brain or rather to the peripheral nervous system, and (e) are adaptive.

In addition to characteristics of the techniques, other factors are relevant to the need for regulation. The context in which the interventions are applied and the degree of vulnerability of the specific patient group will also be relevant to the need for additional legal protections. For example, the existing laws demonstrate concern with the use of NPD in young, incapable, involuntarily hospitalized, or imprisoned people.

The following review of NPD laws should be read with the evolving technological landscape in mind. The current laws raise multiple questions. For example, some laws apply the same restrictive rules to ablative NPD and non-ablative interventions like psychiatric DBS. While both involve surgical risks, they differ in their degree of reversibility and adaptability. Some laws capture only ablative NPD, but appear to leave out psychiatric DBS, which some may view as posing sufficient risks as to require specific legal protections. Although we focus on NPD and psychosurgery laws in this article, we note that an analogous question pertains to externally applied neuromodulation. ECT is subject to specific laws in some jurisdictions (e.g., India; South Australia; Portugal), raising the question as to whether other more novel forms of non-invasive neuromodulation like tES or TMS should also be similarly regulated.

## Existing Laws Governing NPD

In this section, we provide examples of existing laws governing psychosurgery or NPD that are drawn from around the world. Many countries do not have NPD-specific laws, and instead regulate these procedures according to the general legal rules applicable to human subjects research, medicine and medical device regulation. Others have chosen to enact NPD-specific laws that cover some of these matters. Here we present the topics contained in these NPD-specific laws, noting and evaluating the range of approaches that are revealed on each topic.

### Legal Definitions of NPD

Legal definitions are selected for a particular reason—namely to make it clear when the law applies and when it does not. A central question in selecting a legal definition is why legal regulation is required. Once this is determined, the definition can be tailored to try to capture only those situations requiring that regulation and leaving others outside the law. Definitions can be specific and narrow, or more broad and general, with each approach posing particular problems.

One of the challenges with specific definitions is that they are easier to circumvent by selecting procedures that fall outside the definitions. Specific definitions are also risky for rapidly evolving fields, like NPD, as the laws can more quickly become obsolete. One can instead adopt a general definition, but this would bring other problems. First, a general definition tends to capture too much thus requiring the law to include a list of specific exceptions. Overly broad definitions can also harm rapidly evolving fields like NPD by imposing unnecessary regulation on advances that may not need to be regulated as strictly. There are regulatory techniques that allow for updating or clarification, and these vary in their level of bureaucratic delay and difficulty. On the other hand, a cautious pace of regulatory adaptation is not always a bad thing if it helps to avoid error or irresponsible haste, to identify unintended longer-term harms, or to accrue experience that supports beneficial innovation.

Obsolescence is a serious problem for statutes because they are usually difficult and time-consuming to amend. For this reason, many legal systems use delegated or subordinate legislation (often called regulations) which are more easily amended. The legislature delegates the authority to make these regulations to a specified body, setting out the scope of this delegated authority within the statute. For example, the law may state that psychosurgery includes or excludes those interventions listed in a particular regulation, which itself can be amended as needed. This is an approach that might permit a definition of NPD to be more easily changed from time to time, as needed.

#### Existing Legislative Definitions of NPD

The existing laws typically use a three-part definition that describes psychosurgery as a set of (a) techniques used for a set of (b) included indications, but not for a set of (c) excluded indications. An example is offered by the province of Ontario (Canada) [s.49 (2)] (Mental Health Act R.S.O., 1990), which defines psychosurgery as:

“any procedure that, by direct or indirect access to the brain, removes, destroys or interrupts the continuity of histologically normal brain tissue, or that inserts indwelling electrodes for pulsed electrical stimulation for the purpose of altering behavior or treating psychiatric illness, but does not include neurological procedures used to diagnose or treat organic brain conditions, intractable physical pain or epilepsy, if these conditions are clearly demonstrable.”

Ontario's definition thus includes ablative neurosurgery (both incisional and incision-less techniques like radiosurgery) as well as forms of neuromodulation using implants. These techniques count as psychosurgery under the law only where they are used for specified purposes: altering behavior or treating psychiatric illness. They are not included if used to address organic brain conditions, intractable physical pain or epilepsy.

A less common definitional approach is to define the term for the purpose of the statute as any procedure listed in a subordinate regulation. Three jurisdictions that use subordinate regulations in various ways to define NPD are the United Kingdom and the states of Queensland and New South Wales in Australia.

Examples of legal definitions of NPD from around the world are included in Table 1.

**Table 1:** Legislative definitions of NPD.

**Jurisdiction**	**Technique**	**Included indications**	**Excluded indications**	**Legal citation**
**Pacific**				
New Zealand	“Brain surgery” Any surgery or other treatment intended to destroy any part of the brain or brain function	For mental disorder	N/A	Mental Health (Compulsory Assessment Treatment) Act, 1992 Sections 61, 88
South Australia	“Neurosurgery for mental illness” Leucotomy, amygdaloidotomy, hypothalamotomy, temporal lobectomy,^a^ cingulotomy,^b^ electrode implantation in the brain or any other brain surgery for treatment of mental illness by the elimination or stimulation of apparently normal brain tissue	For mental illness	N/A	*Mental Health Act, 2009* Sections 3, 43
Victoria (Australia)	“Neurosurgery for mental illness” (a) any surgical technique or procedure by which one or more lesions are created in a person's brain on the same or on separate occasions for the purpose of treatment; or (b) the use of intracerebral electrodes to create one or more lesions in a person's brain on the same or on separate occasions for the purpose of treatment; or (c) the use of intracerebral electrodes to cause stimulation through the electrodes on the same or on separate occasions without creating a lesion in the person's brain for the purpose of treatment	For the purpose of treatment “treatment” is defined as including steps to remedy a person's mental illness or to alleviate the symptoms and reduce the ill effects of a person's mental illness “treatment” is defined to include neurosurgery for mental illness.	N/A	*Mental Health Act, 2014a* Sections 3, 6
New South Wales (Australia)	“Psychosurgery” (a) the creation of 1 or more lesions, whether made on the same or separate occasions, in the brain of a person by any surgical technique or procedure, (b) the use of electrodes within the brain to produce such a lesion or lesions, whether on the same or separate occasions, or (c) the use on 1 or more occasions of electrodes within the brain primarily for the purpose of influencing or altering the thoughts, emotions or behavior of a person by stimulation through the electrodes without the production of a lesion in the brain of the person	Primarily for the purpose of influencing or altering thoughts, emotions or behavior	Does not include a technique or procedure carried out for the treatment of a condition or an illness prescribed by the regulations for the purposes of this definition. The regulation specifies that “psychosurgery” does not include neurological procedures carried out for the relief of symptoms of Parkinson's disease, Gilles de la Tourette syndrome, chronic tic disorder, tremor, dystonia and epilepsy.	*Mental Health Act, 2007* Section 83 *Mental Health Regulation, 2019* Section 11
Australian Capital Territory	“Psychiatric surgery” Specialized neurosurgery for psychiatric conditions “Neurosurgery” Surgery on the brain of a person for treating a pathological condition of the physical structure of the brain^c^	For psychiatric conditions	N/A	*Mental Health Act, 2015 A2015-38* Section 145
Queensland (Australia)	“Psychosurgery” A procedure on the brain, that involves deliberate damage to or removal of brain tissue, for the treatment of a mental illness. “Non-ablative neurosurgical procedure” A procedure on the brain, that does not involve deliberate	For the treatment of a mental illness	For the purpose of non-ablative neurosurgery, certain conditions do not constitute mental illness: chronic tic, dystonia, epilepsy, Tourette, Parkinson's, tremor, another neurological disorder prescribed by regulation damage to or removal of brain tissue, for the treatment of a mental illness.	*Mental Health Act, 2016* Section 9, 10, Schedule 3 *Mental Health Regulation, 2017* No provisions in this regulation mention psychosurgery or non-ablative neurosurgical procedures.
Fiji	“Psychosurgery” (a) The creation of one or more lesions, whether made on the same or separate occasions, in the brain of a person by any surgical technique or procedure, when it is done primarily for the purpose of altering the thoughts, emotions or behavior of the person; (b) the use for such a purpose of intracerebral electrodes to produce such a lesion or lesions, whether on the same or separate occasions; or (c) the use on one or more occasions of intracerebral electrodes primarily for the purpose of influencing or altering the thoughts, emotions or behavior of a person by stimulation through the electrodes without the production of a lesion in the brain of the person	Primarily for the purpose of altering the thoughts, emotions or behavior of the person;	Does not include a technique or procedure carried out for the treatment of a condition or illness prescribed for the purposes of this definition.	*Mental Health Act, 2010* Section 52
**Asia**				
India^d^	“Psychosurgery” No interventions specified.	For mental illness	N/A	*Mental Healthcare Act, 2017* Section 96
**Europe**				
United Kingdom^e^	“Any surgical operation for destroying brain tissue or for destroying the functioning of brain tissue” and other forms of treatment specified by regulation The additional treatment specified in the Regulation is not relevant here (implantation of hormones to reduce male sexual drive).	For mental disorder	N/A	*Mental Health Act, 1983 1983 c.20* Section 57 *Mental Health (Hospital, Guardianship and Treatment) (England) Regulations, 2008, 2008 No. 1184* Section 27
Scotland	The law sets out rules for “medical treatment” falling within the definition of “certain surgical operations”: any surgical operation for destroying— brain tissue; or the functioning of brain tissue; and such other types of medical treatment as may be specified in regulations for the purposes of this Section. The regulation specifies “deep brain stimulation” which is defined as “the focal modulation of the activity of specific brain regions by direct electrical stimulation delivered by electrodes which are stereotactically implanted in the brain and attached to a programmable control unit inserted in the chest which delivers electrical stimuli. administered repeatedly, over an extended period.” ^f^ The act also specifies special rules for electro-convulsive therapy and “such other types of medical treatment as may be specified in the regulations” (section 237) The regulation goes on to specify transcranial magnetic stimulation and vagus nerve stimulation. These are defined as follows: “Transcranial magnetic stimulation” means the focal modulation of the activity of specific brain regions, by the administration of a changing magnetic field repeatedly, over an extended period “Vagus nerve stimulation” means the intermittent electrical stimulation of the cervical portion of the left vagus nerve by a surgically implanted, programmable electronic device which administers electrical stimuli repeatedly, over an extended period.	“Medical treatment” means “treatment for mental disorder”	N/A	Mental Health (Care Treatment) (Scotland) Act, 2003 Section 234, 329 *Mental Health (Medical treatment subject to safeguards) (Section 234) (Scotland) Regulations, 2005* *Mental Health (Medical treatment subject to safeguards) (Section 237) (Scotland) Regulations, 2005*
Ireland	“Psycho-surgery” Any surgical operation that destroys brain tissue or the functioning of brain tissue	For the purposes of ameliorating a mental disorder.	N/A	*Mental Health Act, 2001* 2001 No. 25. Sections 58.
Portugal	“Intervenção psicocirúrgica” Translation: psychosurgical intervention No further definition given in the statute.	N/A	N/A	*Lei de Saúde Mental Lei No. 36/98* Art 5
**South America**				
Brazil	“A neuropsicocirurgia e quaisquer tratamentos invasivos e irreversíveis para doenças mentais” Translation: neuropsychosurgery and any invasive and irreversible treatments for mental illness No further definition given in the Resolution.	Para doenças mentais Translation: for mental illnesses	N/A	*Resolução and no. 2.057/2013* Art 19, 20.
Chile	“Psicocirugía o cirugía aplicada al tejido cerebral” Translation: psychosurgery or surgery applied to brain tissue No further definition provided in these documents.	con el fin de suprimir o modificar funcionamientos o conductas del paciente Translation: in order to suppress or modify patient functioning or behavior	N/A	*Ley Numero 20.584, Regula los derechos y deberes que tienen las personas en relación con acciones vinculadas a su atención en salud*. 2012 Art. 24 Chile, 1998 Art. 25 Chile, 2002
**North America**				
Alberta (Canada)	“psychosurgery” Any procedure that, by direct or indirect access to the brain, removes, destroys or interrupts the continuity of histologically normal brain tissue, or that inserts indwelling electrodes for pulsed electric stimulation	For the purpose of altering behavior or treating psychiatric illness	But does not include neurological procedures used to diagnose or treat intractable physical pain or epilepsy where those conditions are clearly demonstrable	*Mental Health Act, 2000*, c M-13 s. 1
Saskatchawan (Canada)	“Psychosurgery” Any procedure that by direct access to the brain removes, destroys or interrupts the normal connections of the brain for the primary purpose of treating a mental disorder or involves the implantation of electrodes^h^	For the primary purpose of treating a mental disorder	But does not include neurosurgical procedures designed to treat reliably diagnosed organic brain conditions or epilepsy	*Mental Health Services Act, S.S., 1984-85-86*, c M-13.1 s. 2.
Texas (USA)	“Psychosurgery” A surgical intervention to sever nerve fibers connecting one part of the brain with another, or to remove or destroy brain tissue	With the intent of modifying or altering severe disturbances of behavior, thought content or mood	Surgery for the relief of intractable physical pain or to treat neurological disease or abnormality	*25 Texas Administrative Code §405.103(15), 1993*
California (USA^i^)	“Psychosurgery” Those operations currently referred to as lobotomy, psychiatric surgery, and behavioral surgery and all other forms of brain surgery”	For the purpose of any of the following: (1) Modification or control of thoughts, feelings, actions, or behavior rather than the treatment of a known and diagnosed physical disease of the brain. (2) Modification of normal brain function or normal brain tissue in order to control thoughts, feelings, actions, or behavior. (3) Treatment of abnormal brain function or abnormal brain tissue in order to modify thoughts, feelings, actions or behavior when the abnormality is not an established cause for those thoughts, feelings, actions, or behavior.	Psychosurgery does not include “prefrontal sonic treatment” wherein there is no destruction of brain tissue.	*California Welfare and Institutions Code § 5325(g)* (introduced in the Lanterman-Petris-Short Act, 1967)
Missouri (USA)	“Psychosurgery” (a) Surgery on the normal brain tissue of an individual not suffering from physical disease for the purpose of changing or controlling behavior; (b) surgery on diseased brain tissue of an individual if the sole object of the surgery is to control, change or affect behavioral disturbances, except seizure disorders	In the case of normal brain tissue–for the purpose of changing or controlling behavior. In the case of diseased brain tissue—where the sole purpose is to control, change or affect behavioral disturbances.	In the case of diseased brain tissue, an exception is made for seizure disorders.	*Revised Statutes of Missouri 630.005(27)*
Utah (USA)	“Psychosurgery” A neurosurgical intervention to modify the brain	To reduce the symptoms of a severely ill psychiatric patient.	N/A	*Utah Administrative Code R523-8-5(1)(c), 2015*
Oregon (USA)	“Psychosurgery” Any operation designed to produce an irreversible lesion or destroy brain tissue	For the primary purpose of altering the thoughts, emotions or behavior of a human being.	Does not include procedures which may produce an irreversible lesion or destroy brain tissues when undertaken to cure well-defined disease states such as brain tumor, epileptic foci and certain chronic pain syndromes	*Oregon Revised Statutes § 677.190*

a *A potential ambiguity in the drafting of South Australia's law is that “temporal lobectomy” is used for treatment of epilepsy (Wiebe et al., 2001; Franzini et al., 2019). Its inclusion in the definition of the phrase “neurosurgery for mental illness” suggests that the law means it be included only when it is performed for mental illness, and not epilepsy, which is not today classified as a mental illness. However, any uncertainty in whether to classify a condition as a mental illness or not would create uncertainty about the scope of the law. For greater clarity, some of the other laws explicitly exclude interventions like epilepsy or pain*.

b *The statute actually uses the term “cingulectomy” and not cingulotomy*.

c *Note a possible legal drafting error in this definition. The legal definition of neurosurgery appears to restrict the meaning of neurosurgery to interventions to situations in which physical pathologies are known. This would appear to restrict the meaning of “psychiatric surgery,” which incorporates the term. This is unlikely to be the intended interpretation of the law*.

d *See also the discussion of this law in Doshi et al. (2019)*.

e *The UK Mental Health Act is presently being revised*.

f *Note that DBS programmable control units are not always implanted in the chest (e.g., Garg et al., 2010), revealing the peril of overly precise legislative drafting*.

*^g^In Brazil, the federal Lei 3268/1957 delegates to the Conselho Federal de Medicina (CFM), a self-regulatory body, the authority to regulate the professional practice of physicians and surgeons. The CFM Resolutions govern the practice of medicine in Brazil and have a binding effect for health care professionals*.

h *Note that there is a potential drafting error. The phrase “for the primary purpose of…” appears to qualify lesional interventions but not the implantation of electrodes. This would have the unusual effect of meaning that the implantation of an electrode would fall within the definition whether or not its primary purpose was to treat a mental disorder, whereas lesional interventions would fit within the definition only when the primary purpose was to do so*.

i *Note that California has three slightly different definitions of psychosurgery occurring in five different legislative provisions: Cal. Welf. & Inst. Code § 4503; 17 CCR § 50510; 22 CCR § 76525, Cal.Welf. & Inst.Code § 5325(g), 9 CCR § 836. Only one of these is definitions is presented here*.

#### Common Problems With the Existing Legislative Definitions

A particular problem in some legal definitions of NPD is the exclusion of interventions for “organic brain conditions.” This reflects a problematic philosophical mind-brain dualism. First, if the goal of legislation is to protect vulnerable patients, it is not clear that a patient is any less vulnerable if there is a demonstrable organic or physical cause for their symptoms (e.g., neurodevelopmental conditions, dementia). Second, the nosological distinction between mental/behavioral and organic brain conditions is not a stable one, as the underlying neurobiology of mental disorders becomes better understood. Several psychiatric illnesses are now known to have structural, neurochemical and electrophysiological pathological substrates within the brain. Some conditions like Parkinson's disease are classified as neurological disorders yet involve not just motor symptoms, but also emotional and cognitive symptoms. It is difficult to understand why the treatment of the condition would fall outside NPD laws because of the Parkinson's disease diagnosis, and treatment of similar emotional and cognitive symptoms alone would fall within NPD laws in the absence of such a diagnosis. Finally, the distinction is conceptually muddled to begin with given that the symptoms of mental disorders can be understood simultaneously at the levels of the mind and the brain, and their causes might be a heterogeneous mixture of psychological, environmental, and intrinsic biological factors. Ultimately, this assumed distinction is an inadequate basis for defining the scope of a law governing NPD, and it would be better to return to the main question of why a regulation is required. Then a definition can be developed to ensure that those cases that should be regulated are in fact regulated, and those that should not are not.

Another problem is posed by statutes that limit the scope of NPD to procedures that have the purpose of treating mental illness or disorder. Many laws stipulate a definition of “mental illness” or “mental disorder” for the purposes of the legislation, and this is critical in setting the scope of the law. The legal definitions may not precisely match the main medical nosologies, introducing a source of ambiguity and confusion. An example of the problem is furnished by the legal variation in approach to NPD for addiction and for intractable aggressive behavior associated with certain disorders or syndromes that involve intellectual disability (Micieli et al., 2016). Whether or not interventions in these types of cases would fit within the NPD laws of Victoria (Australia), Ireland and India depends upon the legal definition of mental illness or disorder in their respective statutes. The law of Victoria (Australia) defines mental illness as “a medical condition that is characterized by a significant disturbance of thought, mood, perception or memory” (s. 4). It is unclear, but this definition appears to exclude addiction and aggressivity associated with intellectual disability. Ireland, on the other hand, defines mental disorder in a manner that clearly includes intellectual disability, but likely excludes addiction (s. 3). Finally, India regulates psychosurgery for the treatment of mental illness, which is defined to include addiction but to exclude cognitive disability [s. 2 (s)].

This review also raises questions about the technological obsolescence of the definitions. Some of the more recently updated statutes directly or indirectly regulate interventions like TMS that do not appear to fit within the older statutes, which instead focus on invasive neurosurgery. For example, Scotland does not include TMS in the rules governing psychiatric neurosurgery, but explicitly includes TMS and vagus nerve stimulation within a set of safeguards applicable to electro-convulsive therapy (Reg. 2005 No. 292). If it is advisable to regulate interventions like TMS, statutes that do not address it should be updated.

Another example of the challenge of adequately describing the changing field of neurological interventions is provided by Queensland (Australia), which regulates a class of interventions labeled “non-ablative neurosurgical procedures,” defined as “a procedure on the brain that does not involve deliberate damage to or removal of brain tissue, for the treatment of mental illness” (Sched. 3). The problem with this definition is that it is potentially very broad. Non-invasive forms of neuromodulation via focused ultrasound or TMS are obviously “procedures on the brain.” However, it is unclear whether these non-invasive procedures were meant to be treated as falling within the legal definition of “non-ablative neurosurgical procedures” even though they would appear to fall within the legal definition^2^.

Finally, definitions that include only those interventions that are for the “primary” purpose of treating mental illness [e.g., Saskatchewan (Canada)] also raise legal questions. For example, if neurosurgery was being undertaken to address a non-psychiatric condition but could be performed in one of two ways, one of which could incidentally alleviate a psychiatric condition, should the choice of that method constitute a choice made primarily to treat mental illness? Mallet et al. (2002) describe the unexpected alleviation of long-standing OCD symptoms in two patients treated with DBS for Parkinson's disease. Their report illustrates that it may sometimes be difficult to identify the primary purpose of an intervention when multiple conditions can be addressed simultaneously. It is also difficult to identify a “primary” non-psychiatric purpose because a condition can include a mix of symptoms, some of which are classified as psychiatric and some as neurological in the prevailing dualistic understanding. Bandini et al. (2007) describe the strategy of switching to DBS in patients with Parkinson's disease to allow the reduction of medication that is causing pathological gambling. Since gambling disorder is classified as a mental disorder, would the selection of surgery to reduce that behavior be surgery for the primary purpose of treating mental illness? Ultimately, dualistic concepts and legal language are poorly-suited to our evolving understanding of mental and behavioral conditions.

### Regulations—Prohibitions Applying to Certain Procedures or Populations

Many of the existing laws prohibit certain kinds of NPD altogether, or their use in certain populations. The laws of New South Wales (s. 83) and the Northern Territory in Australia [s.58 (2)] ban psychosurgery regardless of technique or patient population. An example of a non-legal ban is provided by Japan, where the Japanese Society of Psychology and Neurology passed a general resolution against psychosurgery in 1975 during a period of public controversy (Nudeshima and Taira, 2017).

Some laws prohibit NPD for specific populations defined by age, decisional capacity, or legal status (e.g., prisoners or patients who have been involuntarily hospitalized). These prohibitions appear to be based on concerns about patient vulnerability due to incapacity or the voluntariness of consent given the context.

Examples of complete and partial prohibitions on NPD are set out in Table 2. Note that some laws allow NPD but only where additional approval requirements are satisfied (e.g., approval by tribunals, ethics boards or courts) and these are discussed separately in the next section C. In Table 3, we also include other regulations related to emergencies and advance directives, which also affect eligibility for NPD.

**Table 2:** Examples of different forms of prohibitions on NPD.

**Jurisdiction**	**Prohibition**	**Statutory penalty**	**Notes**	**Legal citation**
**Examples of prohibitions on all or some procedures**				
Queensland (Australia)	“Psychosurgery on another person” is prohibited	2 years imprisonment or “200 penalty units” or ~AU$26,000 in 2019.	The law allows “non-ablative neurosurgical procedures” for mental illness (e.g., DBS) with consent and tribunal approval.	*Mental Health Act, 2016* s. 238-241
New South Wales (Australia)	“Psychosurgery” on another person is prohibited	Maximum penalty: “50 penalty units” or ~AU$5500 in 2019.	The definition of psychosurgery includes DBS for psychiatric purposes.	*Mental Health Act, 2007* s. 83
Northern Territory (Australia)	“Psychosurgery on another person” is prohibited.	Maximum penalty: “85 penalty units” or ~AU$13,345 in 2019.	The definition of psychosurgery includes DBS for psychiatric purposes.	*Mental Health Related Services Act, 1998* s. 58
Oregon (USA)	“Performing psychosurgery”	Oregon Medical Board “may refuse to grant, or may suspend or revoke a license to practice” where someone performs psychosurgery.		Oregon Revised Statutes § 677.190
Chile	“Psychosurgery” is only an option for those with major treatment-resistant depression or very severe obsessive compulsive disorder, which have been refractory to treatment of sufficient quantity, frequency and duration using the established therapies that are available in the country		This approach specifies the indications for which psychosurgery is permitted, and so by implication excludes others.	Resolucion 656 s.2
China	Only approved institutions may perform NPD, and only where certain criteria are met. NPD may be performed only for severe and refractory OCD, depression or anxiety disorder, and may not be performed for schizophrenia or related symptomatologies		In addition to the restriction on eligible indications for NPD, the notification also specifies additional eligibility criteria such as “cerebral pathological finding or frequent abnormal brainwave activity.”	Notification regarding improvement of management and related issues in neurosurgery for psychiatric disorders from the General Office of the Ministry of Health Issue (2008) 70.^a^
**Examples of direct and indirect prohibitions on NPD for incapable patients**				
Ontario (Canada)	“Psychosurgery” shall not be performed on a person who is incapable of consenting	Maximum penalty: fine of CA$25,000	This is an example of direct exclusion of the class of incapable people.	Mental Health Act R.S.O., 1990 s. 49, 80
New Zealand	“Brain surgery” shall not be performed for mental disorder on patients without consent and Tribunal approval	Maximum penalty: fine NZ$500 unless another penalty is specified elsewhere.	New Zealand's law indirectly excludes incapable people by making it clear that first person rather than substitute consent is required.	*Mental Health (Compulsory Assessment Treatment) Act, 1992*. s. 61, 121
Connecticut (USA)	“Psychosurgery” shall not be performed without written informed consent by a patient in “any inpatient or outpatient hospital, clinic or other facility for the diagnosis, observation or treatment of persons with psychiatric disabilities.”		This law offers another example of the indirect exclusion by insisting on first person rather than substitute consent. Note, however, that written informed consent is valid for 30 days, revocable at any time, raising the possibility of relying on earlier capable consent after a patient loses capacity.	Connecticut General Statutes § 17a-543(c)
India	“Psychosurgery” shall not be performed as a treatment for mental illness without informed consent of the patient and Board approval.	Maximum penalty for first contravention: Six months imprisonment and/or fine of 10,000 rupees.Subsequent contraventions: Up to 2 years imprisonment and/or a fine of 50,000 to 500,000 rupees.	This law indirectly excludes incapable people by requiring informed consent of the patient.	*Mental Healthcare Act, 2017* s. 96, 108
**Examples of prohibitions for children or minors**				
New Zealand	“Brain surgery” for mental disorder shall not be performed on any person under the age of 17 years	Maximum penalty: fine $500 unless another penalty is specified elsewhere.	Note that this law prohibits NPD for minors under 17 years regardless of whether they have capacity.	*Mental Health (Compulsory Assessment Treatment) Act, 1992*. s. 88
California (USA)	“Psychosurgery” shall in no circumstances be performed on a minor			Cal. Welf. & Inst. Code § 5326.6.
Western Australia	“Psychosurgery” shall not be performed on a person under 16 years of age.	Penalty: 5 years imprisonment.		*Mental Health Act, 2014b* s. 206, 207
South Australia	“Neurosurgery for mental illness” may only be performed if a patient is 16 years of age or older	Maximum penalty: $50,000 or 4 years of imprisonment	This law provides that written consent must be given by the patient, or by the Tribunal if the patient is incapable.	*Mental Health Act, 2009* s. 43
Ontario (Canada)	“Psychosurgery” shall not performed on a person who is incapable of consenting	Maximum penalty: fine of $25,000	The Ontario law is silent regarding minors. Given that the “mature minor doctrine” contemplates that minors may be capable to make treatment decisions, some capable minors but not incapable minors will be eligible in Ontario.	Mental Health Act R.S.O., 1990 s. 49
**Examples of prohibitions applicable to other classes of person (e.g., prisoners, involuntary psychiatric patients)**				
Canada	“Psychosurgery” may not be imposed upon an unfit accused person under the *Criminal Code*.		The Criminal Code of Canada allows a court to direct that an unfit accused person be treated to render them fit to stand trial. Psychosurgery is excluded from these orders.	*Criminal Code of Canada* s. 672.58, 672.61
Newfoundland & Labrador(Canada)	“Psychosurgery” shall not be performed on an involuntarily hospitalized patient.			*Mental Health Care and Treatment Act* SNL 2006 c M-9.1 s. 36

a *Wu et al. (2012) have published a translation of this government document*.

**Table 3:** Examples of types of additional rules regarding eligibility for NPD.

**Jurisdiction**	**Rule**	**Legal citation**
**Rules for advance directives**		
Western Australia	The informed consent of the patient is required for psychosurgery, in addition to Tribunal approval. An adult can give informed consent within an advance directive issued under the *Guardianship and Administration Act 1990*.	*Mental Health Act, 2014b* s. 208 (note 1)
Georgia (USA)	The powers that may be given to a surrogate in an advance directive for health care exclude the ability to consent to psychosurgery.	Ga. Code Ann. § 31-32-7.
**Rules regarding emergencies**		
United Kingdom	In the case of “urgent treatment,” the patient consent and independent approval requirements for NPD are lifted. Four different rules are provided, depending upon the level of medical urgency and the degree of risk and permanence of the intervention. There is no consent or independent approval required if the intervention is immediately necessary to save life. If the intervention is reversible and it is immediately necessary to prevent a serious deterioration in the patient's condition, then consent and independent approval are not required. If the intervention is reversible and non-hazardous and it is immediately necessary to alleviate serious suffering, then consent and independent approval are not required. If the intervention is reversible and non-hazardous and it is immediately necessary to prevent the patient from behaving violently or being a danger to himself or others, then consent and independent approval are not required.	*Mental Health Act, 1983*, 1983 c.20 s. 62
Western Australia	“Psychosurgery” cannot be provided without patient consent in emergencies. Informed consent is not required for “emergency psychiatric treatment.” However, the law states explicitly that “emergency psychiatric treatment” does not include “psychosurgery.”	*Mental Health Act, 2014b* s. 202(2), 203

#### Issues Raised by Legal Prohibitions on NPD for Vulnerable Patient Populations

It is an important ethical problem that the categorical exclusion of vulnerable groups can be both beneficial and harmful at the same time. Exclusion protects them from coerced or otherwise improper treatment. However, it also denies them access to a range of potentially beneficial treatments that others are permitted to have. The 1977 US Commission noted this problem, suggesting that “fairness requires that individuals should not be denied access to potentially beneficial therapy simply because they are involuntarily confined or unable to give informed consent” (page 64). At the same time, the Commission noted their vulnerability to coercion and the possibility that psychosurgery might be proposed to modify their behavior for social or institutional purposes that may not coincide with the patients' own interests or desires (page 65). The Commission proposed that this tension be resolved by adopting a range of protections. First, court review of individual applications should be required. Second, no psychosurgical procedure should be provided to these vulnerable groups until a national psychosurgery advisory board had determined that the procedure showed demonstrable benefit for the psychiatric symptom or disorder.

The solution of limiting NPD to capable patients until there is clear evidence of its safety and efficacy would be workable for some applications of NPD, but might not assist for problems that are encountered primarily in incapable persons (e.g., disorders of consciousness, aggressivity associated with intellectual disability). For example, self-injurious and aggressive behavior co-occurs sometimes with cognitive disability in some conditions (Arron et al., 2011). DBS and ablative NPD are being actively explored to address these aggressive behavioral issues (Gouveia et al., 2019). Research into forms of NPD that are prohibited legally in some places may instead proceed in countries with less restrictive legal environments, although those countries may have alternative forms of oversight that are adequate (e.g., strong research ethics systems and good checks and balances in the surrogate consent rules). However, migration of research to jurisdictions without regulation or an adequate alternative could be a problem if regulatory oversight is in fact warranted. In addition, given the rarity of these procedures, systematic detailed data collection and sharing (with adequate privacy protection) on every single case is important. As a result, it would be better to encourage research on conditions associated primarily with such vulnerable populations to occur in jurisdictions where proper oversight, data collection and publication of results can be ensured. Jurisdictions currently lacking that oversight should work toward developing these mechanisms so that local research may be supported and participants protected.

This remains an important tension to navigate today in light of the legislated restrictions on the books, particularly for forms of NPD that have a good benefit to risk ratio and that are used voluntarily by capable patients.

### Regulations—Independent Approval Procedures

Multiple jurisdictions impose independent approval procedures in addition to informed consent. These procedures vary in three main ways. First, they vary in who must provide the independent review and approval (e.g., independent physicians, government-appointed physicians or laypeople, hospital ethics committee, specialized administrative tribunal or courts). Examples of all of these are provided in Table 4. Second, the procedures differ in the amount of detail specified about what the independent body is expected to verify and the conditions for the body's approval. Third, they vary in whether the same independent approval process is set for all cases, or different approval processes are required for certain patient populations (e.g., prisoners, children, incapable patients).

**Table 4:** Examples of independent review procedures.

**Jurisdiction**	**Approval body or individual**	**Approval process and requirements**	**Legal citation**
**Examples of approval by independent individuals**			
Scotland (Approval where the patient is capable. See below for the approval process where the patient is incapable).	Independent medical practitioner and two people appointed by the Commission who are not non-medical practitioners.	Capable patients: An independent designated medical practitioner must certify in writing that the patient is capable, consents, and the treatment is in the patient's best interests. If the patient is a child, this practitioner must be a child specialist if the patient's responsible physician is not a child specialist. Two people appointed by the Commission who are not medical practitioners must certify in writing that the patient is capable and has consented.	*Mental Health (Care Treatment) (Scotland) Act, 2003* s. 235
United Kingdom	Independent appointed medical practitioner and two other appointees who are not medical practitioners.	The appointed independent medical practitioner and two other non-medical appointees must certify in writing that the patient is capable and has consented. The appointed independent medical practitioner must also certify in writing that the treatment is appropriate. Before doing so, the practitioner must consult with two people (other than the responsible clinician) who have been professionally involved with the patient's treatment. One must be a nurse and the other must be neither a nurse nor a medical practitioner.	*Mental Health Act, 1983*, 1983 c.20 s. 57
California	Three qualified physicians One must be appointed by the facility and two by the local mental health director. Two must be psychiatrists or neurosurgeons.	Three independent physicians must personally examine the patient and unanimously agree with the attending physician that the patient has capacity to consent, that all other appropriate treatments have been exhausted, psychosurgery is definitely indicated, and psychosurgery is the least drastic alternative available for the patient.	Cal. Welf. & Inst. Code 5326.6
Portugal	Psychiatrists appointed by the National Council of Mental Health	Favorable written opinion of two appointed psychiatrists.	*Lei de Saúde Mental Lei No. 36/98* Art. 5.
Chile	Independent psychiatric opinion	In addition to consent, the treating psychiatrist must complete a pre-operative protocol that specifies multiple medical details and is signed by an independent psychiatrist who confirms the treating physician's recommendation. As discussed below the file must also be sent for prior approval by the National Commission for the Protection of Persons Affected by Mental Illness	*Decreto 570* Art. 25 *Resolucion 656* s. 2, 3 *Ley* Art. 14, 15, 24
**Examples of approval by specialized bodies (courts, mental health tribunals, medical commissions, ethics review boards)**			
Western Australia	Mental Health Tribunal	In addition to informed consent by the patient, the psychiatrist must submit an application in writing for Tribunal approval. The application must contain reasons for recommending psychosurgery as well as a detailed treatment plan. The law specifies the conditions for Tribunal approval.	*Mental Health Act, 2014b* 2014 No. 024. Section 208, 384, 416-421.
Victoria (Australia)	Mental Health Tribunal	In addition to informed consent by the patient, the psychiatrist must apply for approval for approval to the Tribunal, which must hear the application and decide within 30 business days.	*Mental Health Act, 2014a* Section 100-103, 152-153
China	Medical Ethics Committee	Approval from the Medical Ethics Committee is required in each case of neurosurgery for psychiatric disorders.	Notification regarding improvement of management and related issues in neurosurgery for psychiatric disorders from the General Office of the Ministry of Health Issue (2008) 70.^a^
Ireland	Mental Health Tribunal Court	Adult patient: In addition to written consent by the patient, the responsible psychiatrist must notify the Mental Health Commission in writing of the proposed psychosurgery, and the Commission refers the matter to the Tribunal. An appeal to the Court. Is available from the Tribunal decision. Child patient: Psychosurgery may not be performed on a child who has been involuntarily hospitalized without Court approval	*Mental Health Act, 2001*, 2001 No. 25. s. 19, 25(12), 48-49, 58
Brazil	Technical Chamber of Psychiatry and plenary session of the Regional Council of Medicine Judicial body in certain cases	In addition to the informed consent of the patient or legal guardian of the patient, the Chamber must prepare an opinion for approval by the Regional Council of Medicine. The Chamber may request advice from professionals in fields related to medicine in forming its opinion. The Regional Council of Medicine must consider and approve the Chamber's opinion in a plenary session. If the patient is involuntarily or compulsorily hospitalized, then prior judicial authorization is required.	Resolução and no. 2.057/2013, 2013 Art. 19
India	Mental Health Review Boards (constituted by the State Mental Health Authority under s. 73)	Approval from the relevant Mental Health Review Board is required to perform psychosurgery for mental illness.	*Mental Healthcare Act, 2017* s. 96
Chile	Comisión Nacional de Protección de las Personas Afectadas de Enfermedades Mentales Trans: National Commission for the Protection of Persons Affected by Mental Illness.	In addition to consent, the psychiatrist must complete a pre-operative protocol, containing a confirmatory opinion from an independent psychiatrist. The medical file must be sent to the Commission a minimum of 30 days before the proposed psychosurgery, in order for the Commission to verify that the patient satisfies eligibility criteria for psychosurgery set out in the Resolution and also to ensure the protection of the patient's rights.	*Decreto 570* Art. 25 *Resolucion 656* Section 3.3 *Ley* Art. 14, 15, 25
Scotland (Approval process where the patient is incapable. See above for the approval process where the patient is capable).	Independent medical practitioner, two people appointed by the Commission who are not non-medical practitioners and the Court of Session.	Incapable patients: Psychosurgery may be provided to an incapable patient who is not objecting to the treatment as long as an independent designated medical practitioner and two appointees who are not medical practitioners have approved and the Court of Session has approved An independent designated medical practitioner must certify in writing that the patient is incapable, does not object, and the treatment is in the patient's best interests. If the patient is a child, this practitioner must be a child specialist if the patient's responsible physician is not a child specialist. Two people appointed by the Commission who are not medical practitioners must certify in writing that the patient is incapable and does not object. The Court of Session may approve if it is satisfied that the patient does not object and the treatment is in the patient's best interests.	*Mental Health (Care and Treatment) (Scotland) Act, 2003* s. 236.

a *Wu et al. (2012) have published a translation of this government document*.

The *Mental Health Act (**2014b**)* of the state of Western Australia is an example of a regulation that includes considerable detail of the decision-making process and approval criteria to be applied by the Mental Health Tribunal. The patient's psychiatrist must apply to the Tribunal in writing, setting out (a) the reasons for recommending psychosurgery, and (b) the treatment plan for psychosurgery including a detailed description of the proposed psychosurgery, the name, qualifications and experience of the proposed neurosurgeon, and the name and address of the place where the psychosurgery is to be performed (s. 417). Parties to the proceeding before the Mental Health Tribunal include the patient, the applicant psychiatrist, and any other person the Tribunal feels has a sufficient interest in the matter to be included (s. 418). The Tribunal cannot approve unless it is satisfied of five things: the patient has given informed consent, the psychosurgery has clinical merit and is appropriate, all reasonably available alternative treatments likely to offer sufficient and lasting benefit have been tried without success, the proposed neurosurgeon is suitably qualified and experienced, and the proposed place for the performance of the psychosurgery is suitable (s. 419). The Tribunal must take into consideration a list of matters in reaching its decision on whether to approve the psychosurgery: (a) the views of any caregiver, close family member or personal support person of the patient, (b) the consequences for the treatment and care of the patient of not performing the psychosurgery, (c) the nature and degree of risk of the psychosurgery, (d) whether the psychosurgery is likely to promote and maintain the health and well-being of the patient, and (e) any other things the Tribunal regards as relevant to the decision (s. 420). Many other laws provide little or no detail of this kind, although the consideration of many of these matters is implicit in performing the function of an independent review tribunal.

Several jurisdictions apply different types of independent approval requirements depending upon the class of patient^3^. Court approval is required in Ireland when the patient is a child who has been involuntarily hospitalized [s. 25 (12)]. In Brazil, judicial approval is required if the patient is involuntarily or compulsorily hospitalized (Art. 19). In Scotland, court approval is required in the case of incapable patients [s. 236 (4)].

#### Advantages and Disadvantages of Approval Procedures

Independent review procedures have advantages and disadvantages. They can protect against improper conduct by clinicians or institutions by ensuring independent scrutiny of cases before NPD is provided. Clinicians and institutions might welcome this independent scrutiny as providing external assurance of the appropriateness of the treatment in a clinical context that is potentially controversial. Another advantage of specialized mental health tribunals is that they can ensure multidisciplinary input into decisions by regulating the composition of the tribunal, specifying the professional expertise of independent appointees, and requiring consultation of a broad range of individuals. On the other hand, additional approval procedures increase the burden on patients and clinicians, and also raise the possibility that capable patients may be refused a treatment that they want and that their own clinicians, who know them better than the review bodies, are recommending. Attendance by the treating physicians at the review body discussions may help to ensure that decisions are adequately informed.

The foregoing are all examples of mandatory independent review and approval procedures. It is worth noting that advisory, as opposed to mandatory, review procedures may also work. For example, in Belgium, the Flemish Stereotactic Neurosurgery for Psychiatric Disorders (SNPD) Committee includes members from the four unversities of Flanders and was established in the 1970s to review proposals for psychiatric neurosurgery, to ensure the appropriateness of the treatment and the patient's ability to give informed consent (Cosyns et al., 1994; Gabriëls et al., 2008). Its initial role was advisory, although its approval now appears to be legally required in at least some situations (Belgium, 2016).

### Regulations—Data Collection Requirements

Some jurisdictions require that periodic reports on the performance of NPD be submitted to a government body or government-appointed individual. The legal reporting requirements vary in detail, some including patient information and others merely reporting on the number and type of procedures performed. The less detailed reports offer less valuable opportunities for oversight. Examples of reporting requirements are provided in Table 5.

**Table 5:** Examples of regulatory reporting requirements.

**Jurisdiction**	**Content**	**Reporting chain**	**Citation**
California (USA)	Quarterly report of the number of involuntary, voluntary, capable and incapable patients who receive psychosurgery Quarterly report of any records or information from reports indicating violation of the law or regulations	Any doctor or facility administering psychosurgery and the State Department of State Hospitals must send quarterly statistics on numbers of patients to the local mental health director, who then transmits a copy to the Director of Health Care Services. The quarterly report of violations of the law must be sent by the Director of Health Services to the Medical Board of California	Cal.Welf. & Inst.Code § 5326.15
Texas (USA)	Quarterly report indicating the number of voluntary and involuntary patients who received psychosurgery as well as the number of involuntary patients for whom a guardian consented, the age, sex and race of the patients, the source of the treatment payment, as well as autopsy findings if death occurred within 14 days of the treatment. Other information required by Departmental rule must also be included.	Any doctor, mental hospital or facility that administer psychosurgery must submit quarterly reports to the department. The department is entitled to use this information to analyze, audit and monitor the use of psychosurgery. The department will file an annual report with the governor and the presiding officer of each house of the legislature summarizing this information.	Health & Safety Code § 578.007 and 578.008 25 TAC § 405.112
Western Australia	As soon as practicable, a report of the performance of psychosurgery in each individual case and a copy of the Mental Health Tribunal approval of the procedure. Annual statistics on performance of psychosurgery.	Patient's psychiatrist must report to the Chief Psychiatrist, and if the patient is a mentally impaired accused, the psychiatrist must also report to the Mentally Impaired Accused Review Board. Chief Psychiatrist must report annual statistics to the Minister, who must report to Parliament.	*Mental Health Act, 2014b* Section 209, 533-534
Victoria (Australia)	Written report to the Chief Psychiatrist on the results of the neurosurgery for mental illness within 3 months after the surgery and within 9–12 months after the surgery is performed.	The psychiatrist who applied for Tribunal approval or the treating psychiatrist must report to the Chief Psychiatrist, who may require further information.	*Mental Health Act, 2014a* Section 104
China	Provincial health administrative departments must inspect and supervise NPD, and provide a summary of previously performed NPD to the Ministry of Health.	The provincial health administrative department must report to the Ministry of Health	Notification regarding improvement of management and related issues in neurosurgery for psychiatric disorders from the General Office of the Ministry of Health Issue (2008) 70.^a^

a *Wu et al. (2012) have published a translation of this government document*.

Some laws regulate the data that must be included in medical charts. For example, California requires that the attending and treating physician(s) place a signed statement in the patient's treatment record of the reasons for the procedure, the fact that all other appropriate treatment modalities have been exhausted, that psychosurgery is definitely indicated and is the least drastic alternative available for the treatment of the patient [California, Cal. Welf & Inst. Code § 5326.6(c)]. In addition, three additional physicians (one must be appointed by the facility and two appointed by the local mental health director; and two must be psychiatrists or neurosurgeons) must personally examine the patient and agree with the attending physician's assessment of the patient's capacity to consent as well as with the appropriateness of the psychosurgery. This must be documented and signed by them in the treatment record [California, Cal. Welf & Inst. Code § 5326.6(d)].

#### Advantages and Disadvantages of Reporting Requirements

Regulatory reporting requirements offer a useful way to ensure that adequately detailed information is collected about what remains an infrequent and exceptional form of intervention, and it also offers a means of retrospective oversight. It is worth noting that experts in the field have argued for more data collection and sharing, viewing it as “crucial to realizing the potential of a number of neurotechnologies and their use in clinical practice” (Ramirez-Zamora et al., 2019). The 1977 US National Commission report recommended that a mechanism be created to collect data about: the nature, extent and outcomes of psychosurgical procedures performed in the USA, the indications for the procedures, and the populations on which they are performed.

One downside of this type of data collection is the invasion of patient privacy, and the risk that highly sensitive patient records might be inadvertently compromised. The US National Commission noted this risk, recommending that stringent privacy safeguards be used (United States National Commission for the Protection of Human Subjects of Biomedical Behavioral Research, 1977).

### Experimental Forms of NPD

In this article, we have not reviewed the rules applicable to experimental NPD procedures. Many forms of NPD remain experimental, and so additional legal rules pertaining to when it is acceptable to attempt treatments that are not within the accepted standard of care will apply, in addition to the NPD-specific laws canvassed here. For example, where the regulatory regime prohibits psychosurgery for minors, any concurrent human subjects research rules that allow medical research in minors should not be taken to suggest that psychosurgery research in minors is permitted.

Most of the NPD-specific laws do not distinguish between experimental treatment and established clinical treatments, and will apply to both. A couple of laws do address the issue of whether the proposed NPD is an established treatment modality or not. For example, Chile (2002) Decree on Psychosurgery justifies strict regulations of psychosurgery on the basis of a general lack of scientific evidence, lack of consensus about the possible benefits and harms, and international ethical controversy. It allows psychosurgery only for severe treatment-resistant depression or OCD.

## Regulatory Variation and the International Movement of Patients

An issue that may arise as a result of variation in the NPD laws is that research activity and patients may move to jurisdictions that are more permissive. This possibility is illustrated by the tendency, documented by the former Psychosurgery Review Board of the state of Victoria in Australia,^4^ for patients to move from Australian states that prohibit psychosurgery to those that permit it. The 2011–2012 report of the Psychosurgery Review Board observed that:

“One of the applications received in 2011/12 concerned an individual from South Australia. This continued a pattern of previous years, in that five applications since 2007 have related to the treatment of individuals from interstate. This occurs because of restrictions or prohibitions on the availability of deep-brain stimulation for the treatment of mental illness in other Australian jurisdictions. New South Wales–where psychosurgery is banned–is examining this…” (Victoria Psychosurgery Review Board 2011/2012 Annual Report Melbourne:, 2012, p. 26).

Widespread migration of patients between countries seems unlikely at the moment given the expense and investigational status of much NPD. However, one can still find advertisements for medical tourism that mention ablative surgery, DBS and vagus nerve stimulation for psychiatric disorders, as well as a range of established functional neurosurgical procedures for other conditions (Neurosurgery in Mexico, 2020). Indeed, medical tourism for established neurosurgical treatments may occur due to lack of availability or cost in the home country (e.g., Idowu and Adewole, 2015). In addition, patients may migrate between jurisdictions after they have received treatment, raising questions about access to ongoing care in their new countries.

To the extent that movement of patients to access NPD is occurring, an important issue relates to how to ensure appropriate follow-up and management of patients such as those with implanted DBS. Furthermore, migration would be troubling if the regulations in the receiving jurisdictions are inadequate. This is an important issue that should be monitored. It also suggests that governments and professional associations should take steps to harmonize regulatory standards and to ensure their enforcement.

## Conclusion

This review of some of the NPD-specific laws enacted around the world reveals that diverse legal jurisdictions have viewed this area of medical intervention as warranting specific regulatory attention, and that this view persists today. Indeed, many laws have been recently enacted or amended particularly in countries in Asia, Europe, and Australia. The kinds of matters contained in these laws vary, but they usually relate to restrictions on eligibility for NPD, specialized approval procedures, and data collection and reporting requirements.

Substantial legislative challenges surround drafting appropriate laws in a context of rapid evolution in technology and medical practice. The challenge is not just one of defining the scope of the regulation appropriately, but even of identifying where regulation is presently required or may in the future be required.

Authoritative voices within the medical community see a need for consensus guidelines and some have called for mandatory regulation, as well as for greater data collection and sharing in this field. Work should now continue to answer the questions set out in the introduction to this article. First, is there a need for specific guidelines or rules addressing NPD? Multiple jurisdictions have decided that specific rules, rather than rules that apply generally to all medical research and practice, are needed for NPD. It is worth noting that this does not always have to take the form of formal legislation, but might emerge from self-regulatory initiatives by the profession.

A significant challenge will be to decide what forms of intervention ought to be regulated, and to select a legal definition that can survive change in technique and practice over time. Our review has revealed how difficult this is, and suggests that the best approach is reflected by jurisdictions such as Scotland that do not attempt to exhaustively list the specific interventions to be regulated within the statute, but instead indicate that in addition to listed interventions, other types of treatment may be specified in regulations from time to time. This method or some version thereof would allow the system to be updated to add or remove types of interventions as the need for regulation becomes clear.

Another critical question is what the regulations should say. Multidisciplinary reflection on how best to promote and protect the interests of a vulnerable group of patients will be essential. Harms may flow from improper interventions but also from laws that exclude people from treatments. An important dimension for international reflection is how to handle the migration of research and patients among jurisdictions with different rules and oversight mechanisms. Finally, systems that allow for more robust data collection and sharing will help to protect patients and to advance the field, and a key role international regulatory harmonization could be to achieve this objective, in addition to addressing the potential risks of medical tourism for NPD.

We have not analyzed the suitability for NPD of existing systems of oversight and regulation of human subjects research or the possible inconsistencies between those systems and existing NPD laws. This should also be pursued in future work. Nevertheless, in our view, available information is sufficient to conclude that appropriate regulations are essential to the safe and ethical development of a promising field that may offer an option to patients with severe and intractable suffering.

## Author Contributions

JC researched, wrote and revised the manuscript. All other authors provided regional knowledge of relevant law and medical practice, and reviewed and revised the manuscript.

## Conflict of Interest

JF receives book royalties for Rights Come to Mind: Brain Injury, Ethics and the Struggle for Consciousness from Cambridge University Press. CHa was part of an unrelated advisory board for Medtronic. BK is a consultant with Medtronic and Abbott, has fellow funding from Medtronic, Abbott and Boston Scientific, and holds an MJFF grant. HM receives consulting and intellectual licensing fees from Abbott Neuromodulation. BN has implanted devices, which were generously provided by Medtronic, for deep brain stimulation (DBS) in patients suffering from obsessive compulsive disorder (OCD). Medtronic provided BN grants for research, education and traveling. BN held the Medtronic Chair for Stereotactic Neurosurgery in Psychiatric Disorders at KU Leuven as well as a Chair for Neuromodulation, an endowment from Medtronic. BN co-owns a patent on DBS in OCD. AJO-M is recipient of a grant from Schuhfried GmBH for norming and validation of cognitive tests, and is national coordinator for Portugal of a Non-interventional Study (EDMS-ERI-143085581, 4.0) to characterize a Treatment-Resistant Depression Cohort in Europe, sponsored by Janssen-Cilag Ltd, and a trial of psilocybin therapy for treatment-resistant depression (EudraCT NUMBER: 2017-003288-36), sponsored by Compass Pathways Ltd. The remaining authors declare that the research was conducted in the absence of any commercial or financial relationships that could be construed as a potential conflict of interest.

## Footnotes

^1^Utah Code § 17-43-308. This code provision remains intact after this section was amended in 2018. An interesting problem of legal interpretation arises in the case of mental disorders displaying religiously-oriented symptoms. Would treatment of the condition run afoul of the criminal legal restriction?

^2^Laws do not typically offer illustrative examples to aid in interpretation. However, the Queensland statute cites DBS as an example of what is meant by the phrase “non-ablative neurosurgical procedure.” This offers some guidance for interpreting the law, but the example still serves to illustrate the potential problem of uncertainty about the precise scope of what constitutes a neurosurgical procedure.

^3^The California Penal Code discusses a court approval procedure for “organic therapy” including psychosurgery for at least some prisoners (California Penal Code §, 2670-2680), although the scope of the provision is confusing and it is inconsistent with a separate legal provision, which seems to prohibit psychosurgery for all prisoners (California Code of Regulations. 15 CCR § 3999.349).

^4^The Psychosurgery Review Board was established under s. 56 of the Victorian *Mental Health Act 1986* to hear applications by psychiatrists for a neurosurgeon to perform psychosurgery on patients. This Board ceased to exist in 2014, when its functions were assumed by the Mental Health Tribunal constituted under the *Mental Health Act (**2014a**)*.
